# Use of an intraoperative navigation system and piezoelectric surgery for styloidectomy in a patient with Eagle’s syndrome: a case report

**DOI:** 10.1186/s13256-017-1464-3

**Published:** 2017-11-15

**Authors:** Shintaro Sukegawa, Takahiro Kanno, Akio Yoshimoto, Kenichi Matsumoto, Yuka Sukegawa-Takahashi, Masanori Masui, Yoshihiko Furuki

**Affiliations:** 1 Division of Oral and Maxillofacial Surgery, Kagawa Prefectural Central Hospital, 1-2-1, Asahi-machi, Takamatsu, Kagawa 760-8557 Japan; 2 Department of Oral and Maxillofacial Surgery, Shimane University Faculty of Medicine, 89-1 Enya-cho, Izumo, Shimane 693-8501 Japan; 3 Yoshimoto Dental Office, 1968-9, Yashimanishi-machi, Takamatsu-shi, Kagawa 761-0113 Japan

**Keywords:** Intraoperative navigation system, Piezoelectric surgery, Eagle’s syndrome, Transoral approach, Case report

## Abstract

**Background:**

Elongated styloid process syndrome (Eagle’s syndrome) is the term given to the symptomatic elongation of the styloid process or the mineralization of the stylohyoid or stylomandibular ligament. The two commonly used approaches for the surgical treatment of this syndrome are the transcervical and transoral approaches. Both have their limitations and specific intraoperative risks. Here, we report the treatment of a patient with Eagle’s syndrome using the transoral approach in conjunction with piezoelectric surgery, surgical planning, and intraoperative navigation to reduce the risk of complications.

**Case presentation:**

The elongated styloid process was resected in a 45-year-old Japanese man using a minimally invasive approach with an intraoperative navigation system. Preoperative preparation involved the use of a custom interocclusal splint to produce the mouth opening conditions required during surgery. Using the three-dimensional position of the navigation probe, the location of the elongated styloid process was identified. After confirmation of the resection spot via the transoral approach, the styloid process was dissected by piezoelectric surgery. Follow-up examination showed an uneventful recovery with no associated complications.

**Conclusion:**

The resection of the styloid process using an intraoperative navigation system and a custom interocclusal splint during a transoral approach, together with a piezoelectric cutting device, is safe and effective for the treatment of Eagle’s syndrome.

## Background

Symptomatic elongation of the styloid process (SP) or the mineralization of the stylohyoid/stylomandibular ligament is termed elongated styloid process syndrome. It is also called Eagle’s syndrome, named after the American physician Eagle, who first reported a series of uncomfortable symptoms, including throat pain and foreign body sensation on the affected side, reflex otalgia, head and neck pain, and hypersalivation, in 1937 [1]. Surgical treatment was selected in instances where no symptomatic improvement was noticed following conservative treatment.

The two different approaches used for the surgical treatment of Eagle’s syndrome are the transcervical and transoral approaches [2]. The cervical approach provides better visualization of the surrounding structures, but has the major disadvantage of an external scar. The transoral approach is commonly accepted due to superior cosmetic results; however, this approach has the disadvantage of compromised intraoperative vision. This may hinder the management of intraoperative complications, such as bleeding, or cause difficulties in the identification of the SP [1, 3]. Surgical treatment is conventionally performed by drilling or cutting with a reciprocating saw and is not free from complications [4]. Therefore, in recent years, several modified methods have been proposed to improve the surgical outcome and reduce morbidity. One report introduced an endoscopically assisted transoral approach to overcome the potential disadvantages of limited exposure and visibility [5]. Another modification method proposed the removal of the SP by combining piezoelectric surgery and a surgical navigation system via the transcervical approach [3].

Here, we present another aspect of the treatment modalities used for Eagle’s syndrome. We report the use of a transoral approach in conjunction with piezoelectric surgery, surgical planning, and intraoperative navigation to reduce the risk of complications.

## Case presentation

**Fig 1:**
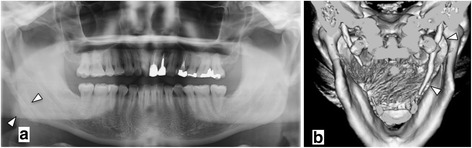
Panoramic radiographs (**a**) and three-dimensional computed tomography (**b**) revealed an elongated styloid process on the right side lateral to the ramus (*arrowheads*)

A 45-year-old Japanese man was referred to our hospital by his local dentist; he had constant pain in the head and neck region, particularly on the right side. The dentist had initially suspected temporomandibular disease, but owing to atypical symptoms, he was eventually referred to our hospital for further examination. He had various symptoms, including dysphagia, foreign body sensation, and headache. Our patient had been treated by several specialists including an otolaryngologist, a neurologist, and another dentist, during the past few years with no relief or signs of improvement. At first presentation, he complained of constant neck pain and odynophagia; however, palpation of the tonsils did not worsen the pain. Our patient had no history of a trauma, surgical treatment, or neurological or infectious foci. After clinical examination and basic radiological diagnostics, an elongated SP was suspected as the cause of his problems. Computed tomography (CT) images showed a longer and thicker right SP when compared with the left side. Our patient was diagnosed with Eagle’s syndrome in view of the neck pain and radiographic images (Fig. 1). Analgesics (daily doses of meloxicam 10 mg) were administered to our patient; the condition was explained to him and operative treatment was recommended. Our patient declined the strong percutaneous approach; therefore, we decided to use the transoral approach. To minimize the risk of damage to the surrounding healthy tissue and difficulty in locating the SP, preoperative digital planning was performed for computed navigation.

**Fig. 2:**
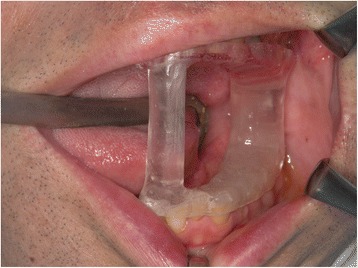
Intraoperative view of the custom interocclusal splint for the maintenance of maximum mandibular opening position. The struts for stabilizing the mandibular position were minimized in view of the surgical operation

An investigation of the cervical blood vessels on the right side demonstrated that the SP and the branches of the internal and external carotid arteries were located in proximity to each other. Therefore, accurate preoperative planning was essential. Furthermore, since the transoral approach was being employed, it was necessary to reproduce the same mouth opening conditions during the taking of preoperative images as that required during surgery. This is because the position of the SP and the blood vessels may change depending on the position of the mandible during the mouth opening. As it is difficult to implement the locational findings from the preoperative imaging data while performing the surgery owing to the mobile nature of the mandible, we used a custom interocclusal splint for repeated maximum opening in the same mandibular position while enabling surgical access. As part of the CT imaging preparation for navigation, a custom interocclusal splint was fabricated by pressing acrylic resin into a dental mold obtained during the first visit. First, the created bimaxillary upper and lower jaw splints were adhered with resin at the maximum mandibular opening position. Because there was the possibility of distortion with fixation of only the molar portion, we added a supplementary small strut to the anterior region without hindering the surgical approach (Fig. 2). Maxillofacial CT and magnetic resonance imaging (MRI) were obtained using the customized interocclusal splint to maintain the mandible in the same position during repeated movements; this was vital to maintain accuracy during the transoral approach.

### Navigation preparation

**Fig. 3:**
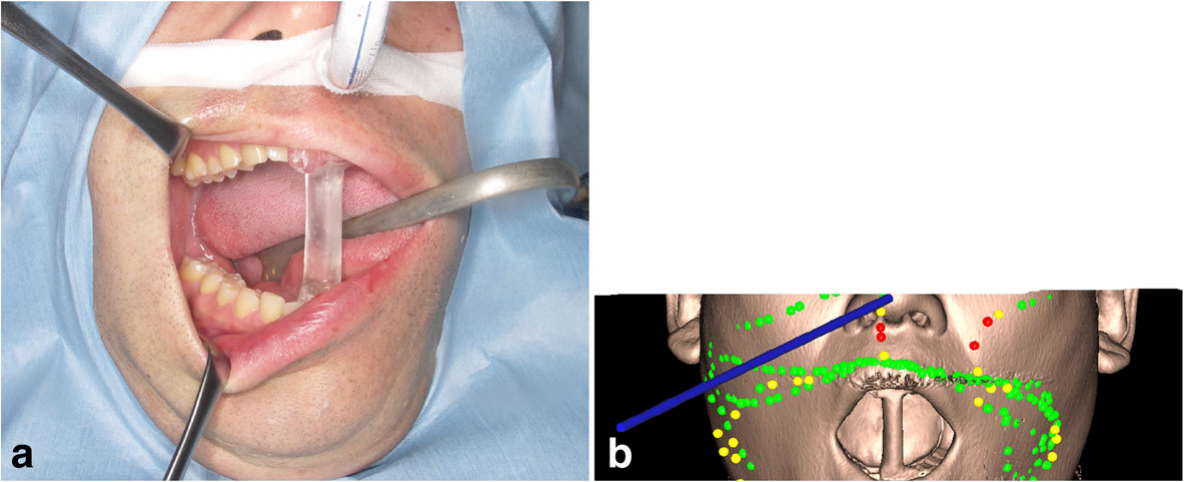
**a** Patient with custom splint during surgery **b** Patient with custom splint was registered using the tracer probe. The *green points* show the trace marked by the probe

The imaging data were obtained in Digital Imaging and Communication in Medicine format and transferred to a Medtronic StealthStation S7 workstation using the Synergy Fusion Cranial 2.2.6 software (Medtronic Navigation Inc., Louisville, CO, USA). Our patient was taken to the operating room where the custom interocclusal splint was reinserted. A Patient Tracker EM, which acted as a reference array to track the navigation probe, was affixed on to our patient’s forehead. To perform patient to CT and MRI data registration, the instrumentation navigation probe was used to trace the reference array and soft tissue landmarks of the face (Fig. 3). After data registration was complete, continuous three-dimensional (3D) tracking of the navigation probe was available to the surgeon in real time. This was possible because of the maximum mandibular opening position during CT and MRI and in the operating room following the use of the interocclusal splint. In this case, as the splint was not in direct contact with the surgical site, it was not sterilized but was chemically disinfected with benzalkonium chloride.

### Surgical procedure

**Fig. 4:**
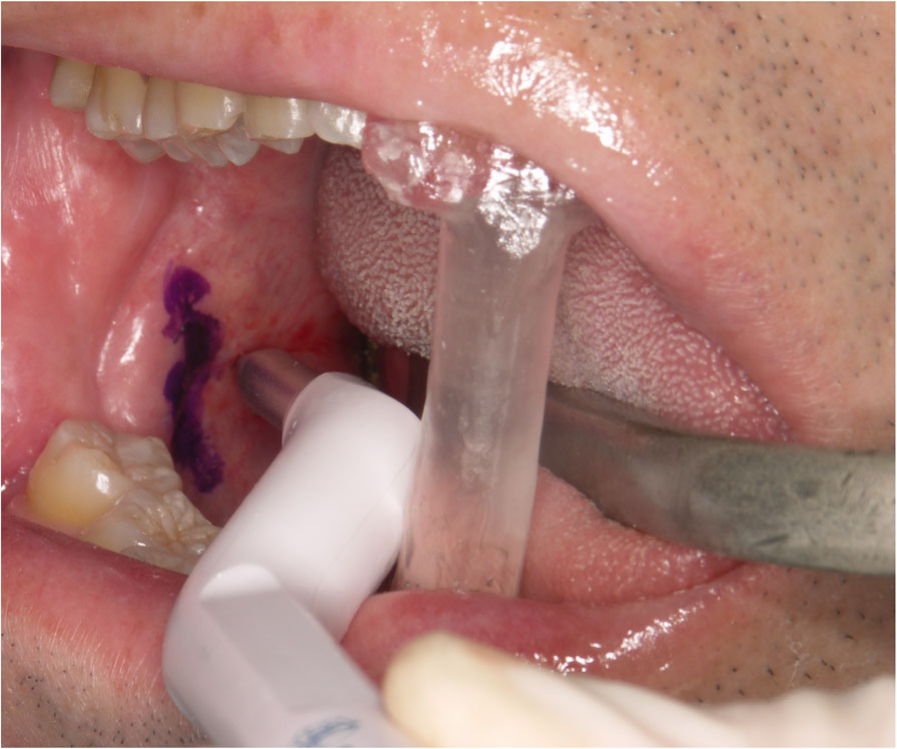
The location of the elongated styloid process was confirmed using a careful navigation system. Setting of intraoral incision line (*purple line*)

**Fig. 5:**
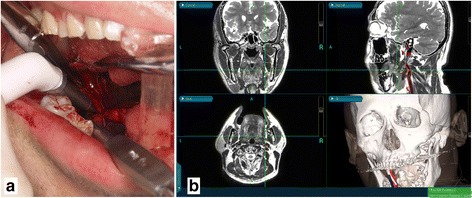
**a** The navigation from the intraoral incision was used to identify the styloid process carefully and positively. **b** Intraoperative navigation system screenshot showing the multiplane view (a fusion of magnetic resonance to computed tomogrpahy images) of the position of the surgeon’s navigation probe in relation to the styloid process and the blood vessels

**Fig. 6:**
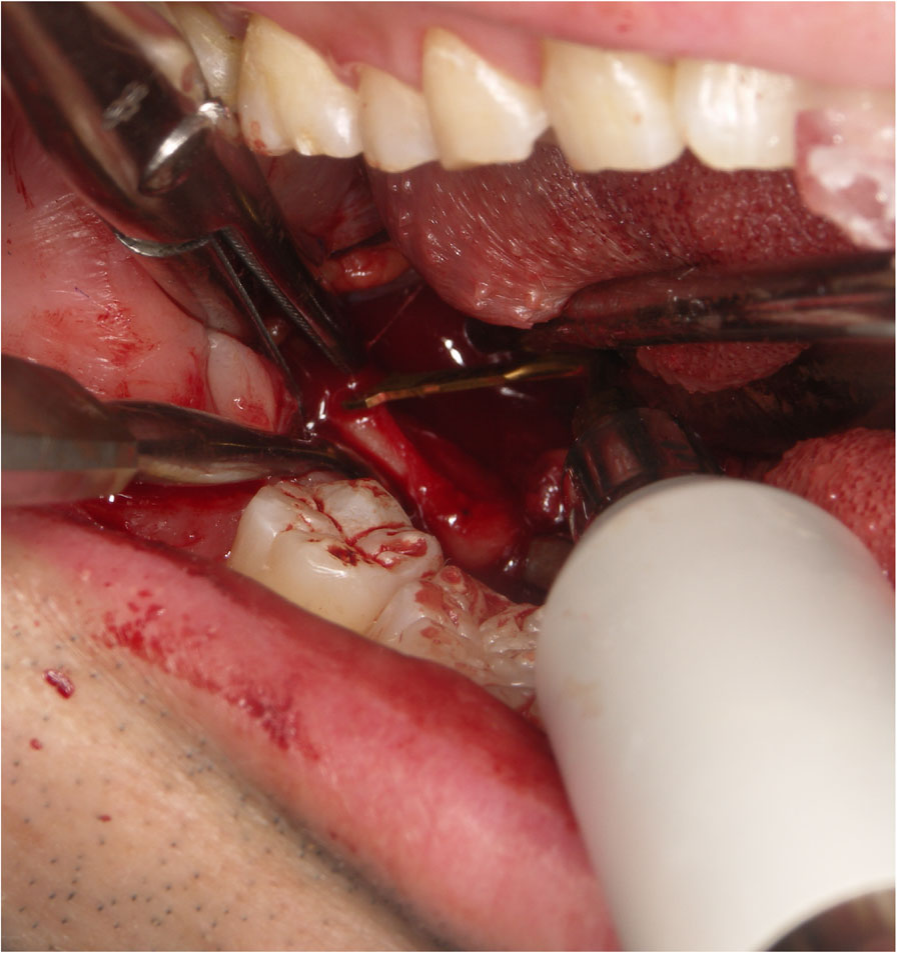
Piezoelectric osteotomies performed at the location of the elongated styloid process confirmed by careful use of the navigation system

**Fig. 7:**
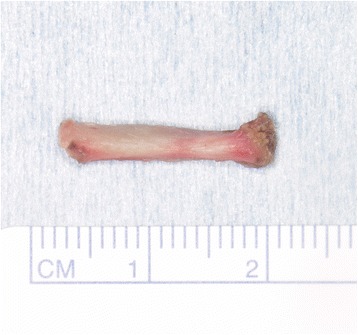
The resected elongated styloid process

Using the navigation pointer, the tip of the elongated SP was detected based on the navigation image on the screen and easily located through the oral mucosa (Fig. 4). The incision in the oral mucosa was designed closest to the tip of the SP to enable easy access with minimal injury. An oral lingual incision (20 mm) was made in the mucosa posterolateral to the pharyngopalatine arch and superior to the tonsillar bed, without tonsillectomy. The blood vessels surrounding the SP were also visible on the navigation monitor, and therefore, we could treat them with care. The navigation pointer was inserted into the wound and moved along the SP until it reached the intended resection spot (Fig. 5). After the resection spot was confirmed, the SP was pinched off using bone-holding forceps while the thicker portion was cut down by piezoelectric surgery (Fig. 6). The resected SP was then taken out (Fig. 7), and a prophylactic drain was left in place before closing the wound on the next day of surgery. The operation time was 37 minutes. The amount of bleeding was very small and it was difficult to measure. Follow-up examination showed an uneventful recovery with no complications. He noticed complete relief of the symptoms on his right side, and he stated after surgery that his quality of life has improved.

## Discussion

The SP is an osseous projection located ventral to the stylomastoid foramen with several ligaments and muscles attached to it. It originates from the tympanohyal and stylohyal segments that are united by cartilage, which often undergoes ossification during middle age [6]. The suspected reasons for SP elongation and calcification of the stylohyoid ligament include pathophysiological mechanisms, such as traumatic fracture of the styloid, compression of the adjacent nerves, degenerative and inflammatory changes in the tendinous portion of the stylohyoid insertion, and irritation of the pharyngeal mucosa by post-tonsillectomy scarring [7]. However, the real reasons for the elongation of the SP and the resulting symptoms remain unclear.

The incidence of elongated SP has been reported to range from 1.4 to 30% [8–10]. However, the incidence of Eagle’s syndrome is much lower with only a small percentage (1 to 5%) of the symptomatic patients [11, 12]. The diagnostic difficulty of this condition is due to the infrequent and vague symptoms associated with it, which can be confused with those of other disorders; thus, conditions such as temporomandibular joint dysfunction, prosopalgia, sphenopalatine neuralgia, glossopharyngeal neuralgia, myofascial pain, mastoiditis, dentalgia, chronic amygdalitis, pharyngitis, and tumors are included in the differential diagnosis [13]. Extreme care is required during diagnosis because misdiagnosis of this syndrome can lead to various unnecessary treatments being administered. Clinical examination and palpation aid in accurate diagnosis. Besides, radiological examinations, such as panoramic radiography and CT, are also very useful. A panoramic radiograph is used to determine whether the SP is elongated [10, 14]. Accurate determination using other two-dimensional (2D) radiographic examinations is difficult due to projection geometry considerations and superimpositions of the mandible as well as the teeth on the SP. Therefore, a panoramic radiograph is effective for the initial diagnosis followed by 3D-CT, which can be used to clearly visualize the structure of the SP and its relationship with adjacent anatomic structures. In addition, it is also used to measure the precise length and angle of the process, which is very significant for the surgery.

The nonsurgical treatment of Eagle’s syndrome generally involves pharmacotherapy with anticonvulsants, antidepressants, and nonsteroidal anti-inflammatory medications; however, the results are short-lived [15]. Lidocaine or steroid is also often injected at the tip of the SP to relieve pain. Nevertheless, pharmaceutical therapy can only provide temporary relief and frequent injections are troublesome. Long-lasting symptomatic relief requires the surgical removal of the long portion of the SP. Transoral and extraoral cervical approaches have proven successful for the surgical management of Eagle’s syndrome to some extent [4, 6]. However, the best surgical approach for this syndrome has not yet been established because both methods are associated with some considerable advantages and disadvantages and should be performed based on the patient’s and surgeon’s circumstances, such as using alternative surgical instruments.

The transcervical approach generally provides an adequate field of operation with access to the SP and its surrounding structures, thus avoiding unnecessary injury to the peripheral neurovascular structures and difficulty in identifying SP. The major disadvantages of this approach include postoperative cosmetic deformity due to scar formation and the requirement of the scope of extensive fascial dissection, which may result in longer duration of surgery and uncomfortable cutaneous nerve paresthesia [2, 15]. Transoral approaches are relatively easy to perform and do not cause postoperative cosmetic deformities; hence, they are considered to be the preferred surgical option. However, the disadvantages of this approach include poor visualization, difficulty in exposing and excising the SP, and the risk of neurovascular injury.

In the present case, preoperative CT revealed a very close relationship between the SP and several branches of the carotid artery. In addition, difficulty due to narrowing of the visual field using the transoral approach was noted. Therefore, we decided to use virtual planning and intraoperative navigation as additional security measures. An intraoperative navigation system is a commonly accepted tool in cranio-maxillofacial surgery. It allows for the safe and fast localization of anatomical structures with minimal exposure [16]. Some authors recommend 3D-CT angiography for better visualization of the blood vessels around the SP [17]. However, by fusing MRI and CT images without radiation exposure, it is possible to perform the surgery while safely observing the blood vessels and soft tissues, such as muscle or fat, in a state reflecting the maximum mandibular opening position. In our opinion, it is also feasible to use intraoperative navigation because of the combined advantages of CT, which helps to easily visualize hard tissues, and MRI, which helps to easily visualize soft tissues.

In the present case, it is important that the mandible position be maintained exactly as that during the surgery to maintain the position of the SP and surrounding blood vessels during mouth opening. As it is difficult to synchronize with the preoperative imaging data during surgery owing to the mobile nature of the mandible, we used a custom interocclusal splint to enable repeated a maximum mandibular opening position while allowing for adequate surgical access. Thereby, a navigation system was further beneficial in determining the accurate location of the object and provided the maxillofacial surgeons with appropriate intraoperative guidance for the safe and reliable use of an individual occlusion splint. We have previously reported the importance of a splint during navigation surgery for mandible lesions [18, 19]. Consequently, in the present case, we were able to perform a more accurate surgery with the aid of the splint, which was used to reproduce the position of the mandible during the surgical procedure.

Piezoelectric surgery utilizes amplified ultrasonic microvibrations as a minute cutting edge to cut bony tissue. This safe cutting device has been proven to be an effective tool for performing bone surgery; it makes the cutting of hard tissue possible through “selective cutting” without encountering necrosis from overheating due to friction and damaging the soft tissues [5, 20]. Selective bone cutting can be performed reliably and precisely with a narrow field of view to protect and avoid damaging the neurovascular and surrounding tissues. The use of a piezoelectric cutting device to cut bone close to the dangerous zone provides additional security when delicate anatomical structures, such as blood vessels, are proximal; this has been recommended by several researchers [19, 20].

## Conclusions

Resection of the SP using an intraoperative navigation system via a transoral approach and a piezoelectric cutting device proved to be very safe and effective for the management and treatment of Eagle’s syndrome.

## Abbreviations

CT: Computed tomography
MRI: Magnetic resonance imaging
